# SUMOylation of Smad2 mediates TGF-β-regulated endothelial–mesenchymal transition

**DOI:** 10.1016/j.jbc.2023.105244

**Published:** 2023-09-09

**Authors:** Qi Su, Xu Chen, Xing Ling, Danqing Li, Xiang Ren, Yang Zhao, Yanyan Yang, Yuhang Liu, Anqi He, Xinjie Zhu, Xinyi Yang, Wenbin Lu, Hongmei Wu, Yitao Qi

**Affiliations:** Key Laboratory of the Ministry of Education for Medicinal Resources and Natural Pharmaceutical Chemistry, National Engineering Laboratory for Resource Developing of Endangered Chinese Crude Drugs in Northwest China, College of Life Sciences, Shaanxi Normal University, Xi'an, Shaanxi, China

**Keywords:** SUMOylation, SENP1, smad2, TGF-β, EndoMT

## Abstract

Endothelial–mesenchymal transition (EndoMT) is a complex biological process in which endothelial cells are transformed into mesenchymal cells, and dysregulated EndoMT causes a variety of pathological processes. Transforming growth factor beta (TGF-β) signaling effectively induces the EndoMT process in endothelial cells, and Smad2 is the critical protein of the TGF-β signaling pathway. However, whether small ubiquitin-like modifier modification (SUMOylation) is involved in EndoMT remains unclear. Here, we show that Smad2 is predominantly modified by SUMO1 at two major SUMOylation sites with PIAS2α as the primary E3 ligase, whereas SENP1 (sentrin/SUMO-specific protease 1) mediates the deSUMOylation of Smad2. In addition, we identified that SUMOylation significantly enhances the transcriptional activity and protein stability of Smad2, regulating the expression of downstream target genes. SUMOylation increases the phosphorylation of Smad2 and the formation of the Smad2–Smad4 complex, thus promoting the nuclear translocation of Smad2. Ultimately, the wildtype, but not SUMOylation site mutant Smad2 facilitated the EndoMT process. More importantly, TGF-β enhances the nuclear translocation of Smad2 by enhancing its SUMOylation and promoting the EndoMT process. These results demonstrate that SUMOylation of Smad2 plays a critical role in the TGF-β-mediated EndoMT process, providing a new theoretical basis for the treatment and potential drug targets of EndoMT-related clinical diseases.

Endothelial–mesenchymal transition (EndoMT) is the biological process by which endothelial cells (ECs) lose their characteristic endothelial phenotype to transition to a mesenchymal cell phenotype (1). EndoMT was initially described in the embryonic heart development, and recent studies have shown that it is also associated with various pathological processes, such as cardiac fibrosis, vascular calcification, atherosclerosis, and cancer (2, 3). EndoMT is essential for vascular remodeling (4) and is directly involved in the development of hypertensive pulmonary disease. Transforming growth factor beta (TGF-β) is the most important regulator of endogenous EndoMT in cardiovascular disease (5). In primary cultured bovine aortic ECs, TGF-β mediates the phosphorylation of a single serine/threonine site in the Smad2 junctional region in a particular manner (6).

Small ubiquitin-like modifier (SUMO) modification (SUMOylation) is an important post-translational modification of proteins in eukaryotes (7). SUMO proteins are structurally similar to ubiquitin and are catalyzed by activating enzyme E1, binding enzyme E2, and ligating enzyme E3 to couple to target proteins. In the steady state, SUMOylated proteins are uncoupled by the uncoupling activity of the sentrin/SUMO-specific protease (SENP) family (8). SUMOylation regulates a range of cellular response processes, including cellular signaling and cellular metabolism, by altering protein macromolecular interactions and intracellular localization or directly altering the SUMO covalent modification activity of the linked protein substrate (9, 10, 11). SUMOylation is a versatile system that regulates multiple biological and pathological processes, including cardiac development (12), myogenesis (13), sudden unexplained death in epilepsy (14, 15, 16, 17), and leukemia (18).

Smad2 is a signal transduction molecule downstream of the TGF-β family of receptors and plays a critical role in TGF-β signaling of epithelial cells (19, 20). TGF-β1 induces C-terminal phosphorylation of Smad2 and Smad3, and the phosphorylated Smad2 and Smad3 bind to Smad4 for nuclear transport. Smad4 is SUMOylated by SUMO1, and Smad4 SUMOylation regulates TGF-β-mediated transcriptional responses. SUMOylation protects Smad4 from ubiquitin–proteasomal degradation and enhances the transcriptional responses of Smad4 (21). SUMOylation of Smad4 alters its subnuclear localization and enhances its stability (22, 23). Ginkgolic acid is a urushiol that exists primarily in the epicarp of *Ginkgo biloba* and improves bleomycin-induced pulmonary fibrosis by inhibiting SUMOylation of Smad4 (24). Previous studies have shown that PIASy regulates TGF-β-mediated signaling by stimulating SUMOylation and the nuclear export of Smad3 (25). However, it remains unknown whether Smad2 is SUMOylated and the role of SUMOylation in TGF-β regulated EndoMT.

Here, we demonstrate that Smad2 is modified by SUMO1 and that SUMOylation of Smad2 plays a critical role in the TGF-β-mediated EndoMT process. Smad2 was modified by SUMO1 at two SUMOylation sites with PIAS2α as the primary E3 ligase, whereas SENP1 mediated the deSUMOylation of Smad2. In addition, SUMOylation significantly enhanced the transcriptional activity of Smad2 and increased the phosphorylation of Smad2 and the formation of the Smad2–Smad4 complex, ultimately promoting the nuclear translocation of Smad2 and the EndoMT process. Together, these results identified the molecular mechanism of Smad2 SUMOylation in EndoMT and provide a new therapeutic target for the clinical treatment of EndoMT-related diseases.

## Results

### Smad2 is SUMOylated by SUMO1 along with the E3 ligase PIAS2ɑ

**Figure 1:**
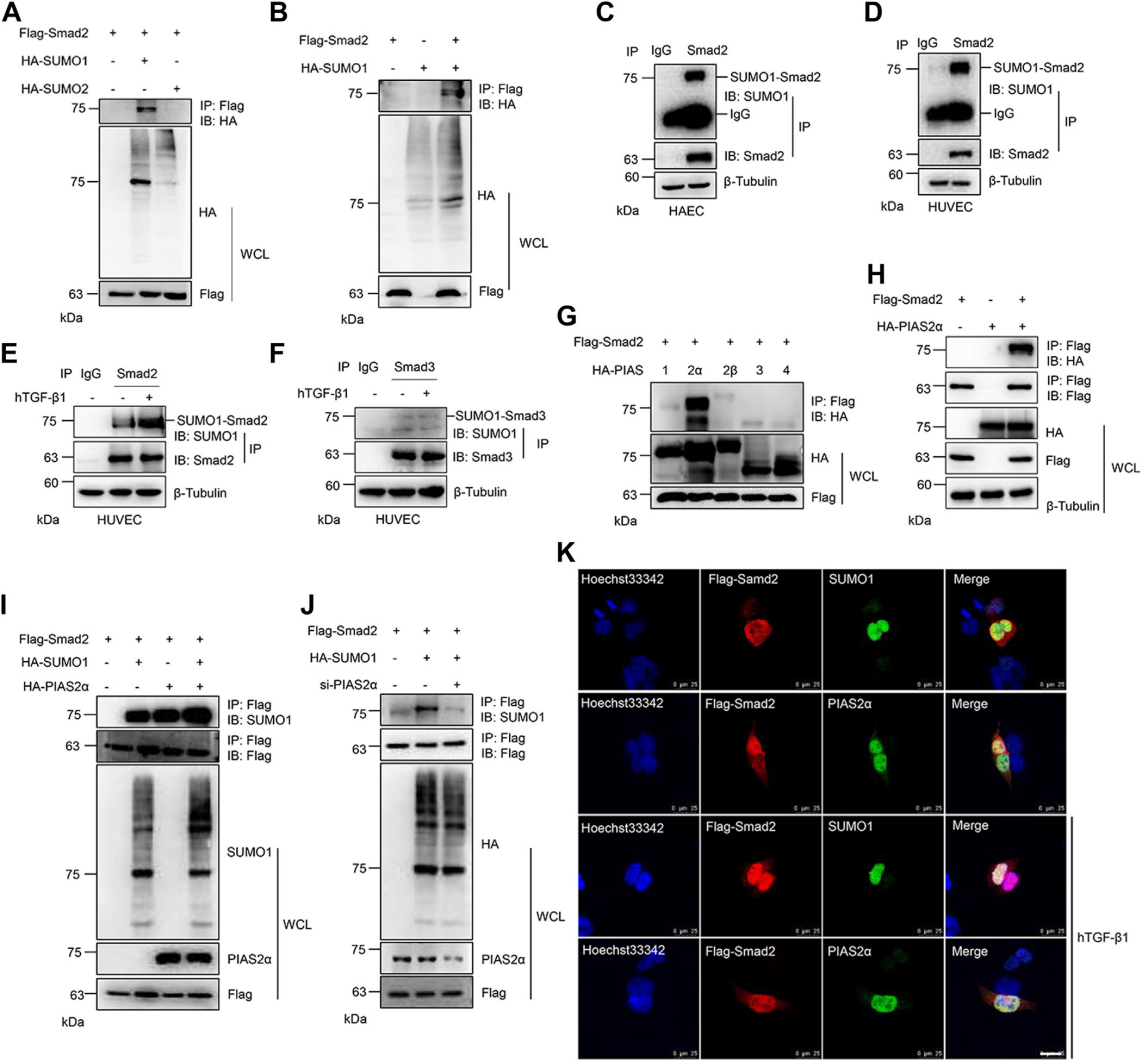
**Smad2 SUMOylation is modified by SUMO1 along with the E3 ligase PIAS2α.***A*, Smad2 was modified by exogenous SUMO1 in HEK293T cells. The indicated plasmids were transfected into HEK293T cells, and the IP with anti-FLAG from cell lysates was detected by IB with anti-HA antibody. The whole-cell lysates (WCLs) were detected by IB with anti-HA or anti-FLAG antibodies. *B*, SUMO1 mediated the SUMOylation of Smad2 in HEK293T cells. FLAG-Smad2 and HA-SUMO1 plasmids were transfected into HEK293T cells, and the IP with anti-FLAG from cell lysates was detected by IB with anti-HA antibody. The WCLs were detected by IB with anti-HA or anti-FLAG antibodies. *C*, Smad2 was modified by endogenous SUMO1 in HAECs. The IP with anti-IgG or anti-Smad2 from HAEC lysates was detected by IB with anti-SUMO1 or anti-Smad2 antibodies. The WCLs were detected by IB with anti-β-Tubulin antibody. *D*, Smad2 was modified by endogenous SUMO1 in HUVECs. The IP with anti-IgG or anti-Smad2 from HUVEC lysates was detected by IB with anti-SUMO1 or anti-Smad2 antibodies. The WCLs were detected by IB with anti-β-tubulin antibody. *E*, TGF-β enhanced the SUMOylation of Smad2 in HUVECs. The cells were treated with hTGF-β1, and the IP with anti-IgG or anti-Smad2 from cell lysates was detected by IB with anti-SUMO1 or anti-Smad2 antibodies. The WCL was detected by IB with anti-β-tubulin antibody. *F*, TGF-β showed no significant effect on the SUMOylation of Smad3 in HUVECs. The cells were treated with hTGF-β1, and the IP with anti-IgG or anti-Smad3 from cell lysates was detected by IB with anti-SUMO1 or anti-Smad3 antibodies. The WCL was detected by IB with anti-β-tubulin antibody. *G*, PIAS2α was the major SUMO E3 ligase of Smad2. The indicated plasmids were transfected into HEK293T cells, and the IP with anti-FLAG from cell lysates was detected by IB with anti-HA antibody. The WCL was detected by IB with anti-HA or anti-FLAG antibodies. *H*, PIAS2α interacted with Smad2. The indicated plasmids were transfected into HEK293T cells, and the IP with anti-FLAG from cell lysates was detected by IB with anti-HA or anti-FLAG antibodies. The WCL was detected by IB with indicated antibodies. *I*, PIAS2α enhanced the SUMOylation of Smad2. The indicated plasmids were transfected into HEK293T cells, and the IP with anti-FLAG from cell lysates was detected by IB with anti-SUMO1 or anti-FLAG antibodies. The WCL was analyzed by IB with the indicated antibodies. *J*, PIAS2α knockdown diminished the SUMOylation of Smad2. HEK293T cells were transfected with the indicated plasmids and si-PIAS2α, and the IP with anti-FLAG from cell lysates was detected by IB with anti-HA or anti-FLAG antibodies. The WCL was analyzed by IB with the indicated antibodies. *K*, Smad2 colocalized with SUMO1 and PIAS2α in the nucleus. The FLAG-Smad2 plasmid was transfected into HEK293T cells or followed by hTGF-β1 treatment. The cells were harvested for immunocytochemistry with anti-FLAG (*red*) and anti-SUMO1 or anti-PIAS2α (*green*) antibodies. DAPI (*blue*) was used to show nuclei. The scale bar represents 25 μm. DAPI, 4′,6-diamidino-2-phenylindole; HA, hemagglutinin; HAEC, human aortic endothelial cell; HEK293T, human embryonic kidney 293T cell line; hTGF-β1, human TGF-β1; IB, immunoblotting; IgG, immunoglobulin G; IP, immunoprecipitate; TGF-β, transforming growth factor beta.

Smad2 is a critical protein in the TGF-β signaling pathway; however, it is unknown whether Smad2 is modified by SUMO. To investigate whether Smad2 was modified by SUMO and which SUMO was responsible for the modification, we transiently transfected human embryonic kidney 293T (HEK293T) cells with FLAG-Smad2 and hemagglutinin (HA)-SUMO plasmids. The coimmunoprecipitation (co-IP) results showed that Smad2 was mainly conjugated with exogenous SUMO1 (Fig. 1*A*), and SUMO1 significantly enhanced the modification of Smad2 (Fig. 1*B*). Next, we examined the endogenous SUMOylation of Smad2 in HEK293T cell and human umbilical vein endothelial cell (HUVEC), and the modification of Smad2 by endogenous SUMO1 was confirmed by a co-IP experiment (Fig. 1, *C* and *D*). Human TGF-β1 (hTGF-β1) also significantly enhanced the SUMOylation of Smad2 in HUVECs (Fig. 1*E*). Although Smad3 is also reported to be SUMOylated (25, 26), hTGF-β1 showed no significant effect on the SUMOylation of Smad3 in HUVECs (Fig. 1*F*). The SUMOylation process is catalyzed by SUMO-specific enzymes, and PIAS family members are the major SUMO E3 ligases. The PIAS and Smad2 plasmids were transiently transfected into HEK293T cells, and the co-IP assay revealed that PIAS2α was the major SUMO E3 ligase that interacts with Smad2 (Fig. 1, *G* and *H*). PIAS2α dramatically enhanced the SUMOylation of Smad2, whereas the knockdown of PIAS2α with si-PIAS2α significantly diminished the SUMOylation of Smad2 (Fig. 1, *I* and *J*), confirming PIAS2α as the primary E3 ligase of Smad2. We further examined the localization of Smad2 with SUMO1 and PIAS2α, and immunofluorescence (IF) staining showed that Smad2 colocalized with SUMO1 and PIAS2α in the nuclear membrane, and hTGF-β1 treatment promoted the colocalization of Smad2 with SUMO1 and PIAS2 in the nucleus (Fig. 1*K*). These results suggested that Smad2 was SUMOylated by SUMO1 with PIAS2α as an E3 ligase.

### K156 and K383 are the major SUMOylation sites of Smad2

**Figure 2:**
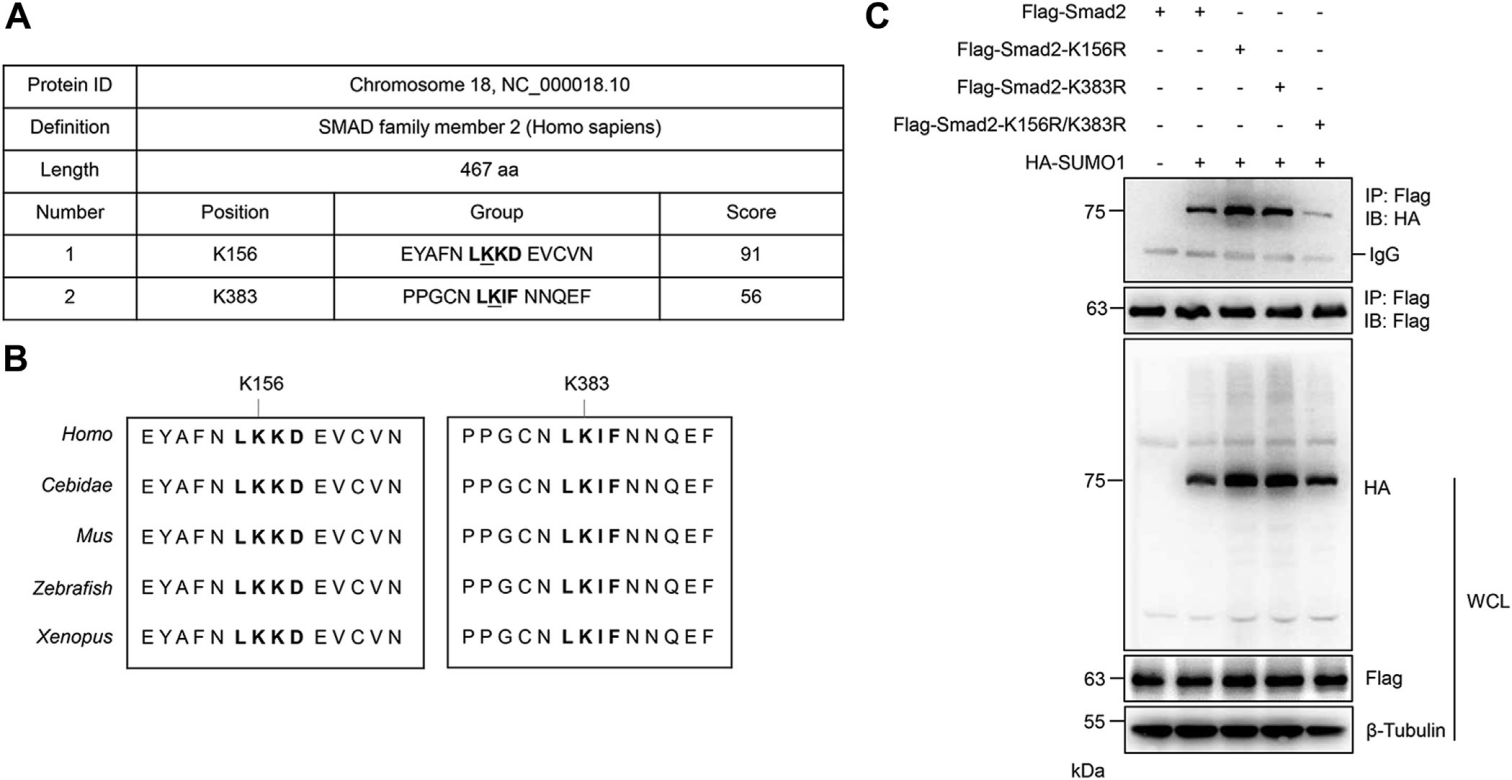
**K156 and K383 are the major SUMOylation sites of Smad2.***A*, the SUMOylation sites of Smad2 were predicted by online software. *B*, the predicted SUMOylation sites K156 and K383 were highly conserved in various species. *C*, K156 and K383 were the major SUMOylation sites of Smad2. Wildtype (w) or SUMO site mutant FLAG-Smad2 and HA-SUMO1 plasmid were transfected into HEK293T cells, and the IP with anti-FLAG from cell lysates was detected by IB with anti-HA antibody. The WCL was analyzed by IB with anti-HA or anti-FLAG antibodies. HA, hemagglutinin; HEK293T, human embryonic kidney 293T cell line; IB, immunoblotting; IP, immunoprecipitate; WCL, whole-cell lysate.

Since Smad2 was modified by SUMO1, the SUMOylation sites of Smad2 were further investigated. The SUMOplot (www.abcepta.com/sumoplot) predicted that Smad2 is highly likely to be SUMOylated, and the bioinformatic analysis of Smad2 revealed two potential SUMO-conjugation consensus sites, K156 and K383 (Fig. 2*A*). These predicted SUMOylation sites are evolutionarily conserved in different species (Fig. 2*B*). We then constructed single SUMOylation site mutation plasmids, including K156R and K383R point mutation plasmids. The co-IP results showed that K156R and K383R had no significant effect on the SUMOylation of Smad2 (Fig. 2*C*). Furthermore, we constructed a double SUMOylation site mutation plasmid, K156R/K383R. The co-IP results showed that K156R/K383R significantly decreased the SUMOylation of Smad2, indicating that K156 and K383 were the major SUMOylation sites of the Smad2 protein (Fig. 2*C*).

### Smad2 is deSUMOylated by SENP1

**Figure 3:**
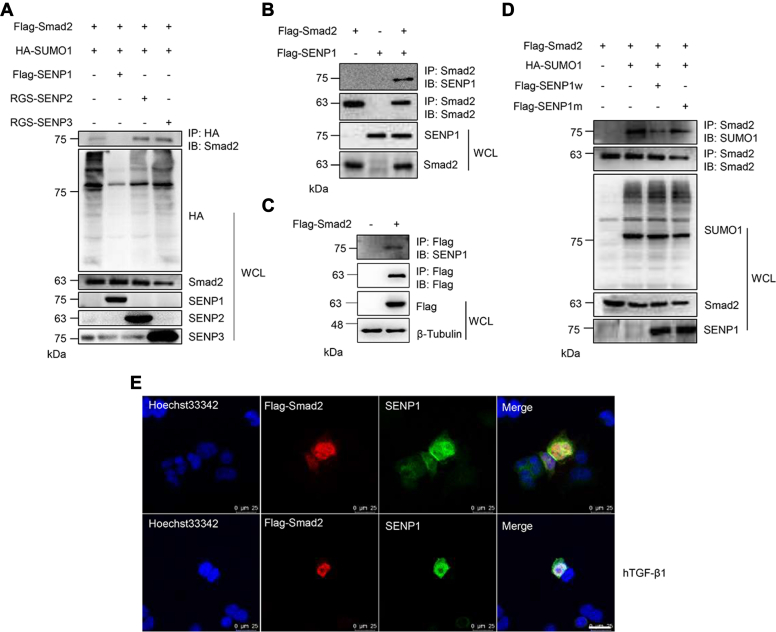
**SENP1 deconjugates SUMOylated Smad2.***A*, SENP1 specifically mediated the deSUMOylation of Smad2. The indicated plasmids were transfected into HEK293T cells, and the IP with anti-HA from cell lysates was detected by IB with anti-Smad2 antibody. The WCLs were analyzed by IB with the indicated antibodies. *B*, Smad2 interacted with exogenous SENP1. FLAG-Smad2 and FLAG-SENP1 plasmids were transfected into HEK293T cells, and the IP with anti-Smad2 from cell lysates was detected by IB with anti-SENP1 or anti-Smad2 antibodies. The WCL was analyzed by IB with anti-SENP1 or anti-Smad2 antibodies. *C*, Smad2 interacted with endogenous SENP1. The FLAG-Smad2 plasmid was transfected into HEK293T cells, and the IP with anti-FLAG from cell lysates was detected by IB with anti-SENP1 or anti-FLAG antibodies. The WCL was analyzed by IB with anti-FLAG or anti-β-tubulin antibodies. *D*, SENP1 deconjugated SUMOylated Smad2 in HEK293T cells. The indicated plasmids were transfected into HEK293T cells, and the IP with anti-Smad2 from cell lysates was detected by IB with anti-SUMO1 or anti-Smad2 antibodies. The WCL was analyzed by IB with the indicated antibodies. *E*, Smad2 colocalized with SENP1 in the nucleus. The FLAG-Smad2 plasmid was transfected into HEK293T cells or followed by hTGF-β1 treatment. The cells were harvested for immunocytochemistry with anti-FLAG (*red*) and anti-SENP1 (*green*) antibodies. DAPI (*blue*) was used to show nuclei. The scale bar represents 25 μm. DAPI, 4′,6-diamidino-2-phenylindole; HA, hemagglutinin; HEK293T, human embryonic kidney 293T cell line; hTGF-β1, human transforming growth factor beta 1; IB, immunoblotting; IP, immunoprecipitate; SENP1, sentrin/SUMO-specific protease; WCL, whole-cell lysate.

SUMOylation is a dynamic process, and de-SUMOylation is regulated explicitly by SENP family members. We determined that Smad2 is mainly SUMOylated by SUMO1 with the E3 ligase PIAS2α, which mediates its SUMOylation. To determine which SENP mediates the deconjugation of SUMOylated Smad2, different SENPs were cotransfected into HEK293T cells, and the co-IP results showed that SENP1, but not SENP2 or SENP3, specifically deconjugated the SUMOylation of Smad2 (Fig. 3*A*). To further identify the interaction of SENP1 with Smad2, both plasmids were transfected into HEK293T cells, and the co-IP results showed that exogenous SENP1 and Smad2 were firmly combined (Fig. 3*B*). Further experiments showed that Smad2 interacted not only with exogenous SENP1 but also with endogenous SENP1 (Fig. 3*C*). Furthermore, wildtype SENP1, but not the catalytic mutant SENP1, deconjugated SUMOylated Smad2 (Fig. 3*D*). IF staining showed that exogenous Smad2 colocalized with endogenous SENP1 in the nuclear membrane, and hTGF-β1 treatment diminished the colocalization of Smad2 with SENP1 in the nucleus (Fig. 3*E*). These results indicated that Smad2 was SUMOylated and SENP1 interacted with Smad2 to deconjugate its SUMOylation.

### SUMOylation enhances the transcriptional activity of Smad2

**Figure 4:**
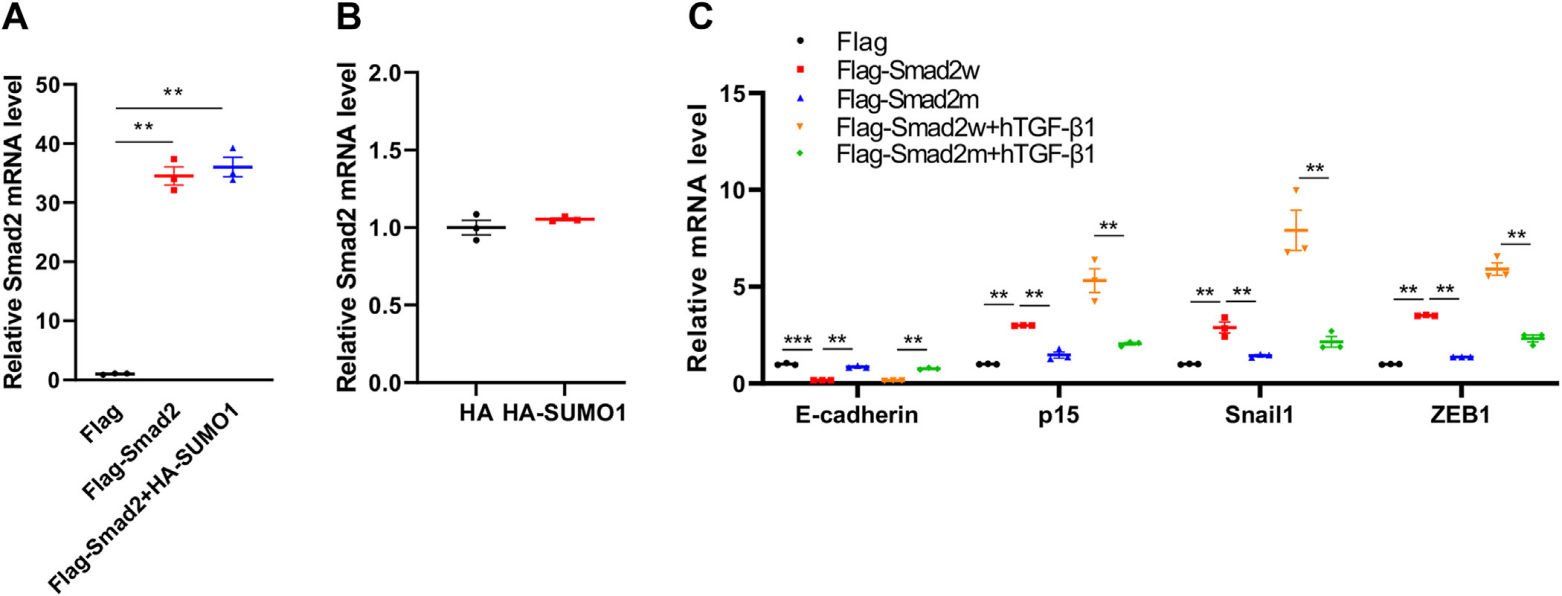
**SUMOylation enhances the transcriptional activity of Smad2.***A*, SUMOylation had no significant effect on the mRNA level of exogeneous Smad2. HEK293T cells were transfected with the indicated plasmids, and the expression levels of Smad2 transcripts were measured by real-time PCR normalized to the control (n = 3 repeats/group). *B*, SUMOylation had no significant effect on the mRNA level of endogeneous Smad2. HEK293T cells were transfected with the indicated plasmids, and the expression levels of Smad2 transcripts were measured by real-time PCR normalized to the control (n = 3 repeats/group). *C*, SUMOylation of Smad2 affected the mRNA level of target genes. HEK293T cells were transfected with the indicated plasmids or followed by hTGF-β1 treatment. The expression levels of transcripts of target genes were measured by real-time PCR normalized to the control (n = 3 repeats/group). HEK293T, human embryonic kidney 293T cell line; hTGF-β1, human transforming growth factor beta 1.

To identify the roles of SUMOylation in Smad2 transcription, FLAG-Smad2 and HA-SUMO1 plasmids were transfected into HEK293T cells, and the mRNA levels of Smad2 were measured. The results did not show a significant variation in exogeneous and endogenous Smad2 mRNA levels in HEK293T cells overexpressing SUMO1 (Fig. 4, *A* and *B*). Next, we examined the effect of SUMOylation on the transcriptional activity of Smad2 by transfecting cells with wildtype Smad2 (Smad2w) *versus* SUMOylation-deficient Smad2. The mRNA levels of downstream Smad2 genes, including E-cadherin, p15, Snail1 (zinc finger transcription factor 1), and ZEB1, were analyzed by RT–quantitative PCR. The results showed that SUMOylation mutation of Smad2 significantly inhibited the transcriptional levels of p15, Snail1, and ZEB1 and promoted the transcriptional level of E-cadherin, whereas hTGF-β1 treatment enhanced the transcriptional activity of Smad2w but diminished the effect of mutant Smad2 (Fig. 4*C*). These results indicated that SUMOylation of Smad2 significantly enhanced its transcriptional activity.

### SUMOylation inhibits the ubiquitin-mediated proteasomal degradation of Smad2

**Figure 5:**
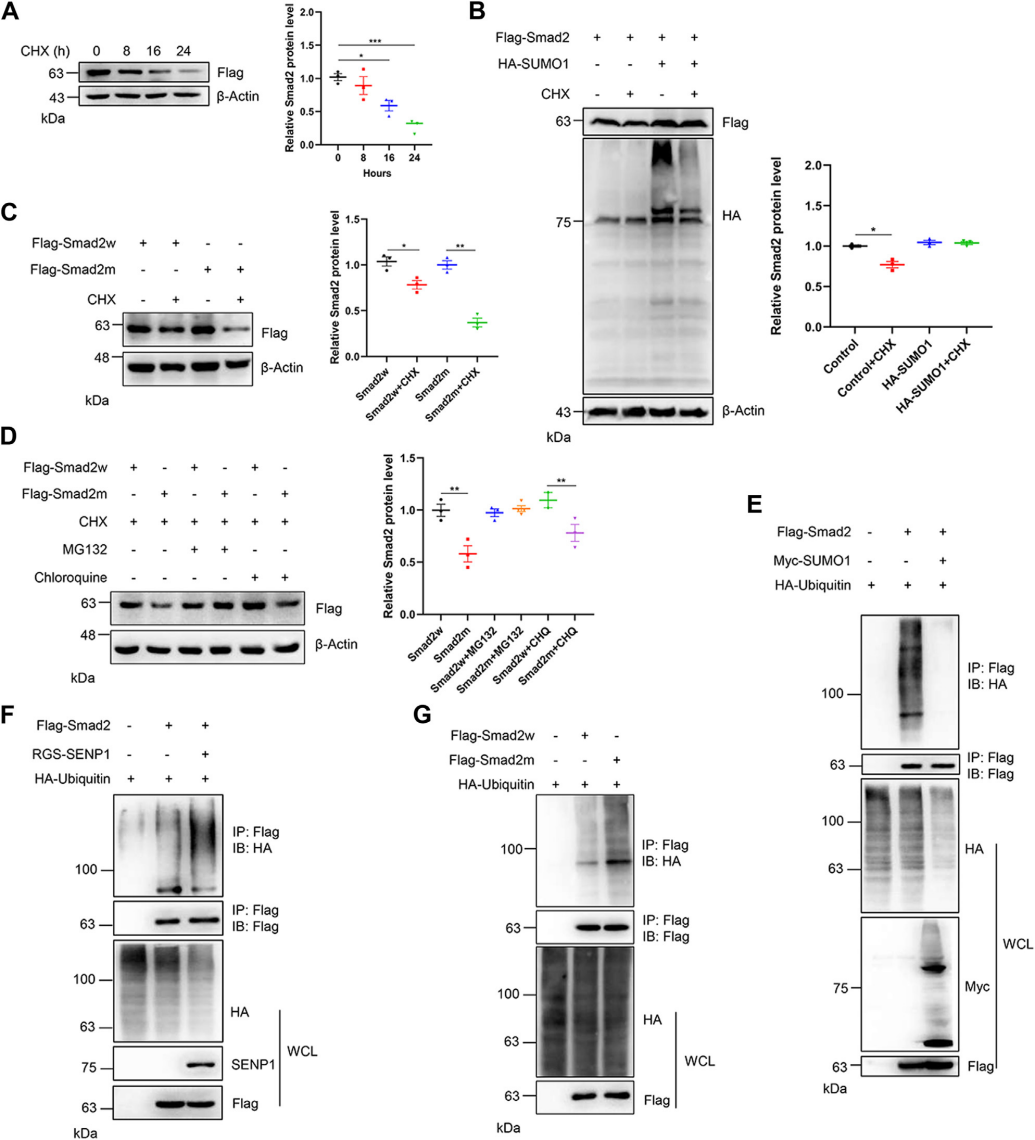
**SUMOylation regulates the stability of Smad2.***A*, Smad2 protein was degraded in a time-dependent manner. HEK293T cells were transfected with the FLAG-Smad2 plasmid and treated with CHX for different periods. The cells were harvested, and the cell lysates were detected by IB with anti-FLAG and anti-β-actin antibodies (*left*). The gray analysis was performed by comparing the FLAG band to the β-actin band (*right*, n = 3 repeats/group). *B*, SUMOylation increased the stability of Smad2. HEK293T cells were transfected with the indicated plasmids, and the cells were treated with CHX for 12 h. The cells were harvested, and the cell lysates were detected by IB with the indicated antibodies (*left*). The gray analysis was performed by comparing the FLAG band to the β-actin band (*right*, n = 3 repeats/group). *C*, SUMO mutation decreased the stability of Smad2. HEK293T cells were transfected with the indicated plasmids, and the cells were treated with CHX for 12 h. The cells were harvested, and the cell lysates were detected by IB with anti-FLAG and anti-β-actin antibodies (*left*). The gray analysis was performed by comparing the FLAG band to the β-actin band (*right*, n = 3 repeats/group). *D*, the decreased expression of Smad2 was rescued by MG132 but not by chloroquine treatment. The transfected HEK293T cells were treated with CHX and followed by treatment with MG132 or chloroquine for 6 h, and the WCLs were harvested and detected by IB with anti-FLAG or anti-β-actin antibodies (*left*). The gray analysis was performed by comparing the Smad2 band to the β-actin band (*right*, n = 3 repeats/group). *E*, SUMO1 overexpression inhibited its ubiquitination. HEK293T cells were transfected with the indicated plasmids, and the IP with anti-FLAG from cell lysates was detected by IB with anti-HA or anti-FLAG antibodies. The WCL was detected by IB with the indicated antibodies. *F*, SENP1 promoted the ubiquitination of Smad2. HEK293T cells were transfected with the indicated plasmids, and the IP with anti-FLAG from cell lysates was detected by IB with anti-HA or anti-FLAG antibodies. The WCL was detected by IB with the indicated antibodies. *G*, SUMO mutation of Smad2 promoted its ubiquitination. HEK293T cells were transfected with the indicated plasmids, and the IP with anti-FLAG from cell lysates was detected by IB with anti-HA antibody. The WCL was detected by IB with anti-HA and anti-FLAG antibodies. CHX, cycloheximide; HA, hemagglutinin; HEK293T, human embryonic kidney 293T cell line; IB, immunoblotting; SENP1, sentrin/SUMO-specific protease 1; WCL, whole-cell lysate.

SUMOylation can positively or negatively regulate protein stability through ubiquitin-mediated proteasomal degradation. HEK293T cells were transfected with FLAG-Smad2, and the protein was harvested after cycloheximide (CHX) treatment for different periods. The results showed that the protein level of Smad2 was significantly reduced after 24 h of CHX treatment (Fig. 5*A*). To investigate the effect of SUMOylation on the stability of Smad2, FLAG-Smad2 and HA-SUMO1 plasmids were transfected into HEK293T cells, and protein was harvested after CHX treatment for 24 h. As shown, SUMOylation significantly inhibited Smad2 degradation (Fig. 5*B*). The Smad2w or the double-SUMO site mutant (Smad2m) plasmid was transfected into HEK293T cells, and the results showed that the blockade of SUMOylation promoted Smad2 degradation (Fig. 5*C*). The transfected cells were treated with MG132 or chloroquine to inhibit the ubiquitin- or lysosome-dependent degradation pathways, and the results showed that MG132, but not chloroquine, prevented the degradation of Smad2 (Fig. 5*D*). To further determine whether SUMOylation affects Smad2 ubiquitination, one of the main pathways of protein degradation, the indicated plasmids were transfected into HEK293T cells, and the results showed that SUMOylation significantly inhibited Smad2 ubiquitination (Fig. 5*E*). In contrast, SUMOylation blockade by SENP1 or SUMO mutation enhanced the ubiquitination level of Smad2 (Fig. 5, *F* and *G*). These results demonstrated that Smad2 was SUMOylated and degraded through the ubiquitin-mediated proteasomal degradation pathway.

### SUMOylation promotes Smad2 phosphorylation and cytoplasm–nucleus transport

**Figure 6:**
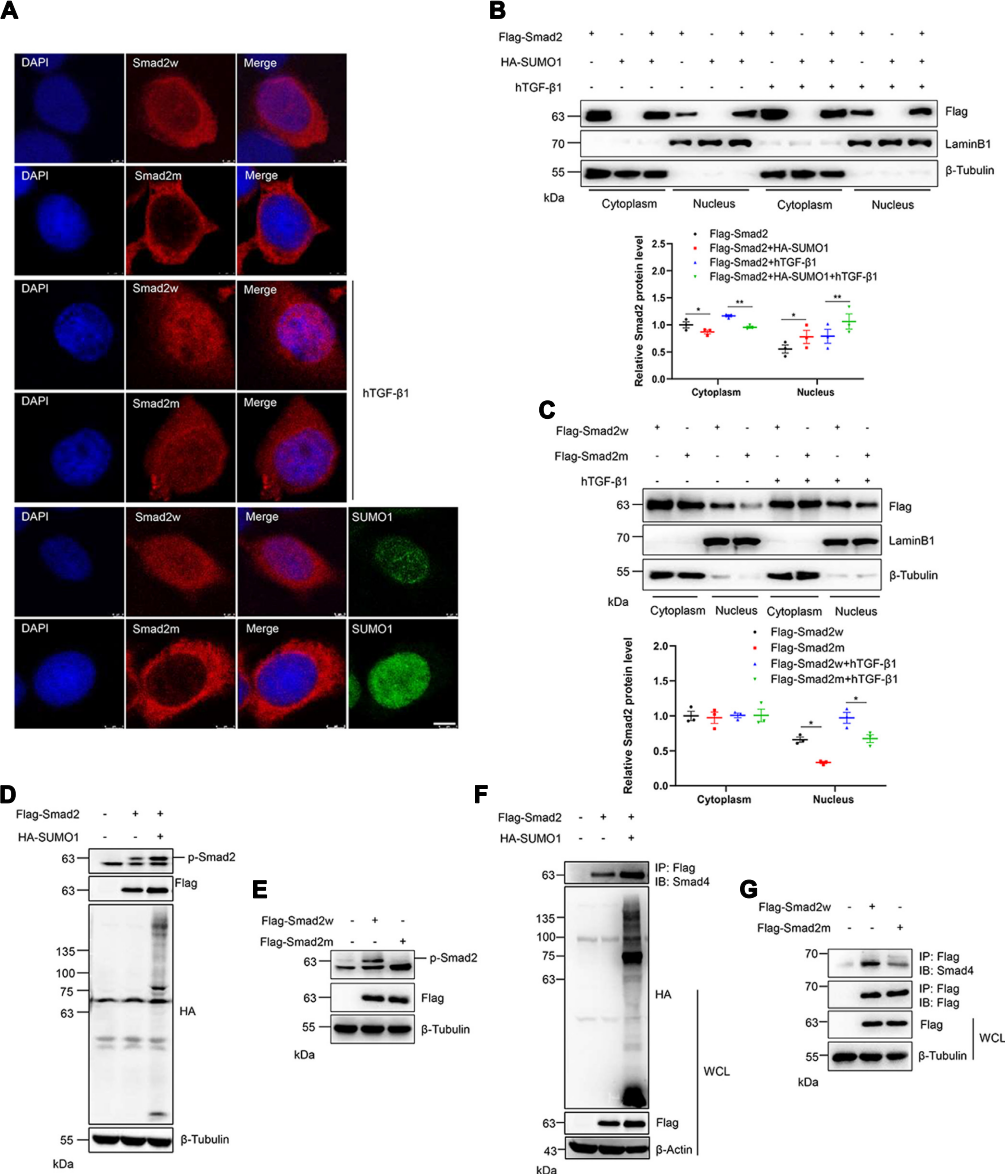
**SUMOylation changes the subcellular localization of Smad2 and promotes the nuclear localization of Smad2.***A*, SUMOylation of Smad2 promoted its localization in the nucleus. Wildtype or SUMO mutant Smad2 with or without HA-SUMO1 plasmid was transfected into HEK293T cells or followed by hTGF-β1 treatment, and the cells were harvested for immunocytochemistry with anti-Smad2 (*red*) and anti-SUMO1 (*green*) antibodies. DAPI (*blue*) was used to show nuclei. The scale bar represents 3 μm. *B*, SUMO1 overexpression promoted its translocation from the cytoplasm to the nucleus. The indicated plasmids were transfected into HEK293T cells and followed by hTGF-β1 treatment, and the lysates from the cytoplasm and nucleus were detected by IB with the indicated antibodies (*upper*). The gray analysis was performed by comparing the FLAG band to the β-tubulin band (*bottom*, n = 3 repeats/group). *C*, SUMOylation site mutant Smad2 inhibited its translocation from the cytoplasm to the nucleus that promoted by wildtype Smad2 (Smad2w). The indicated plasmids were transfected into HEK293T cells and followed by hTGF-β1 treatment, and the lysates from the cytoplasm and the nucleus were detected by IB with the indicated antibodies (*upper*). The gray analysis was performed by comparing the FLAG band to the β-tubulin band (*bottom*, n = 3 repeats/group). *D*, SUMOylation enhanced the phosphorylation level of Smad2. The indicated plasmids were transfected into HEK293T cells, and the cell lysates were detected by IB with the indicated antibodies. *E*, SUMOylation site mutant Smad2 inhibited its phosphorylation level that is enhanced by Smad2w. The indicated plasmids were transfected into HEK293T cells, and the cell lysates were detected by IB with the indicated antibodies. *F*, SUMOylation enhanced the interaction between Smad2 and Smad4. The indicated plasmids were transfected into HEK293T cells, and the IP with anti-FLAG from cell lysates was detected by IB with anti-Smad4 antibody. The WCL was detected by IB with the indicated antibodies. *G*, SUMOylation site mutant Smad2 inhibited the interaction between Smad2 and Smad4 that is enhanced by Smad2w. The indicated plasmids were transfected into HEK293T cells, and the IP with anti-FLAG from cell lysates was detected by IB with anti-Smad4 or anti-FLAG antibodies. The WCL was detected by IB with the indicated antibodies. DAPI, 4′,6-diamidino-2-phenylindole; HA, hemagglutinin; HEK293T, human embryonic kidney 293T cell line; hTGF-β1, human transforming growth factor beta 1; IB, immunoblotting; IP, immunoprecipitate; WCL, whole-cell lysate.

Smad2 was primarily localized in the cytosol, phosphorylated, and interacted with Smad4, ultimately being transported to the nucleus. IF staining showed that Smad2w was preferentially localized in the cytoplasm and nucleus, whereas the SUMO mutant Smad2 remained in the cytoplasm (Fig. 6*A*). Both hTGF-β1 treatment and SUMO1 overexpression enhanced the nuclear localization of Smad2w; however, SUMOylation showed no significant effect on mutant Smad2 (Fig. 6*A*). The fractionation of transfected HEK293T cells also showed that hTGF-β1 treatment and SUMOylation dramatically enhanced Smad2 accumulation in the nucleus (Fig. 6*B*), whereas SUMO site mutation of Smad2 diminished its accumulation in the nucleus (Fig. 6*C*). These results indicated that hyper-SUMOylation facilitated the nuclear import of Smad2 from the cytoplasm. To identify the molecular mechanism of the regulation of SUMOylation in Smad2 transport, the effect of Smad2 phosphorylation by SUMOylation was detected. The results showed that SUMO1 overexpression significantly enhanced the phosphorylation level of Smad2 (Fig. 6*D*), whereas SUMO site mutation decreased the phosphorylation level of Smad2 (Fig. 6*E*). We further examined the binding of Smad2 with Smad4 protein by co-IP, and the results indicated that SUMOylation significantly enhanced the interaction between Smad2 and Smad4, promoting the formation of the Smad2–Smad4 complex (Fig. 6*F*), whereas SUMO site mutation decreased the interaction between Smad2 and Smad4 (Fig. 6*G*). Taken together, these results indicated that SUMOylation significantly enhanced Smad2 phosphorylation, promoted the formation of the Smad2–Smad4 complex, and promoted the translocation of the complex into the nucleus.

### SUMOylation of Smad2 promotes TGF-β-mediated EndoMT

**Figure 7:**
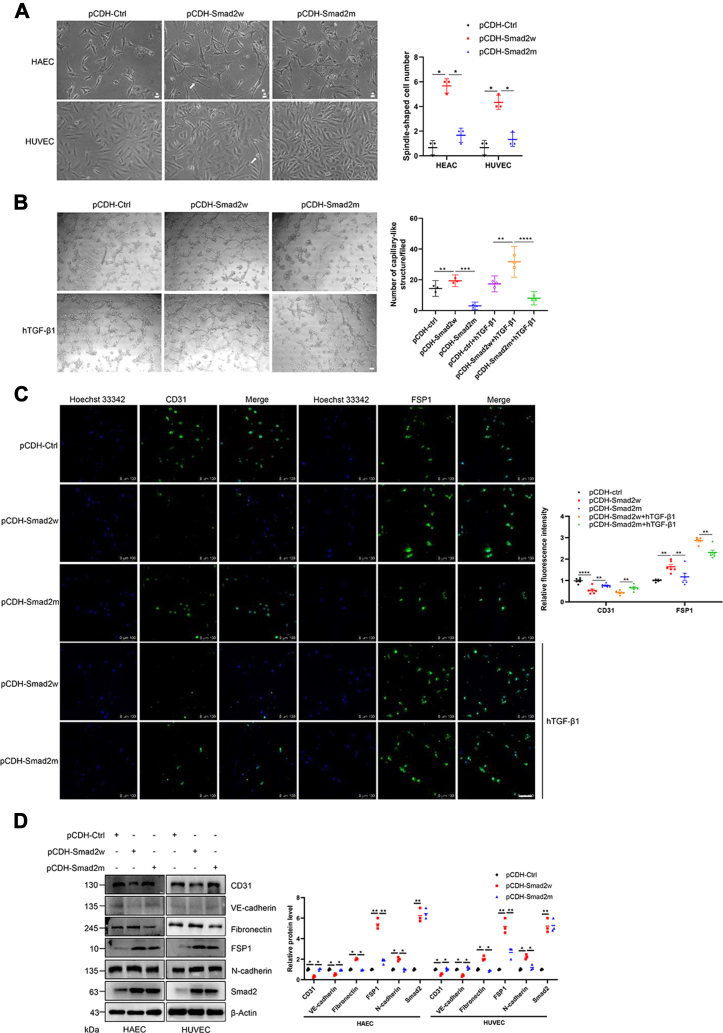
**SUMOylation of Smad2 promotes the TGF-β-mediated EndoMT process.***A*, wildtype but not SUMO mutant Smad2 increased the proportion of spindle-shaped HAECs and HUVECs. Wildtype or SUMO mutant Smad2 stably transfected cells were treated with hTGF-β1 and observed under a light microscope (*left*), and the number of spindle-shaped cells was compared between different groups (*right*, n = 3 repeats/group). The scale bar represents 40 μm. *B*, wildtype but not SUMO mutant Smad2 increased the angiogenic capacity of HUVECs. The HUVECs were stably transfected by wildtype or SUMO mutant Smad2 followed by hTGF-β1 treatment and observed under a light microscope (*left*), and the number of capillary-like structure was compared between different groups (*right*, n = 3 repeats/group). The scale bar represents 100 μm. *C*, wildtype but not SUMO mutant Smad2 increased CD31 and decreased FSP1 in HUVECs. Wildtype or SUMO mutant Smad2 was stably transfected into cells, and the cells were treated with hTGF-β1 and harvested for immunocytochemistry with anti-CD31 or anti-FSP1 (*green*) antibody (*left*). The relative luciferase activity was compared between different groups (*right*, n = 6 repeats/group). Hoechst 33342 (*blue*) was used to show nuclei. The scale bar represents 100 μm. *D*, wildtype but not SUMO mutant Smad2 decreased the expression of endothelial markers and increased the expression of mesenchymal markers in HAECs and HUVECs. Wildtype or SUMO mutant Smad2 was stably transfected into cells. The cell lysates were detected by IB with the indicated antibodies (*left*). The gray analysis was performed by comparing the Smad2 band to the β-actin band (*right*, n = 3 repeats/group). EndoMT, endothelial–mesenchymal transition; FSP1, ferroptosis suppressor protein 1; HAEC, human aortic endothelial cell; hTGF-β1, human transforming growth factor beta 1; HUVEC, human umbilical vein endothelial cell; IB, immunoblotting; TGF-β, transforming growth factor beta.

To further investigate the effect of Smad2 SUMOylation on the TGF-β-mediated EndoMT process, we examined the cytomorphological effect of SUMOylation in ECs by performing EC morphological observation. hTGF-β1 is a potent cytokine that mediates EndoMT and was added to stably expressed Smad2w and SUMO mutant Smad2 human aortic EC (HAEC) and HUVEC lines. Compared with the control group, the Smad2w cells showed spindle-shaped and severe cell elongation, indicating that Smad2w significantly induced the EndoMT process. However, the SUMO mutant Smad2 group showed much less cell fibrosis than the Smad2w group (Fig. 7*A*). These results indicated that the blockade of SUMOylation of Smad2 inhibited the EndoMT morphological formation in ECs. To explore the mechanisms underlying Smad2 SUMOylation in EndoMT, the Matrigel assay was performed to examine the effect of Smad2 SUMOylation on HUVEC tube formation with or without hTGF-β1 treatment. The results showed that Smad2w increased the number of tube-like structures in Matrigel-mixed HUVECs. However, the SUMO mutant Smad2 showed much fewer tube-like structures than Smad2w (Fig. 7*B*). These results suggested that the blockade of SUMOylation inhibited angiogenic functions in ECs.

During the EndoMT process, the morphology of ECs changes gradually, with a decrease in the endothelial-specific proteins, vascular endothelial-cadherin and CD31 (platelet EC adhesion molecule 1), and an increase in the mesenchymal proteins, α-SMA, FSP1 (ferroptosis suppressor protein 1), and N-cadherin. IF staining showed that Smad2w increased the expression of the fibroblast marker FSP1 and reduced the expression of the EC marker CD31 in HUVECs, whereas SUMO mutation of Smad2 weakened its capacity with or without hTGF-β1 treatment (Fig. 7*C*). To further confirm the role of Smad2 SUMOylation in EndoMT, the protein levels of endothelial or mesenchymal markers were examined by Western blot analysis. The results demonstrated that the protein levels of CD31 and vascular endothelial-cadherin were decreased, whereas fibronectin, FSP1, and N-cadherin protein levels were significantly upregulated by Smad2w (Fig. 7*D*). However, cells transfected with the mutant Smad2 construct exhibited much less variation (Fig. 7*D*). Taken together, these results suggested that the mutation of the Smad2 SUMOylation sites reduced the capacity of Smad2 to induce EndoMT in ECs, indicating that the SUMOylation of Smad2 plays a crucial role in EndoMT.

## Discussion

**Figure 8:**
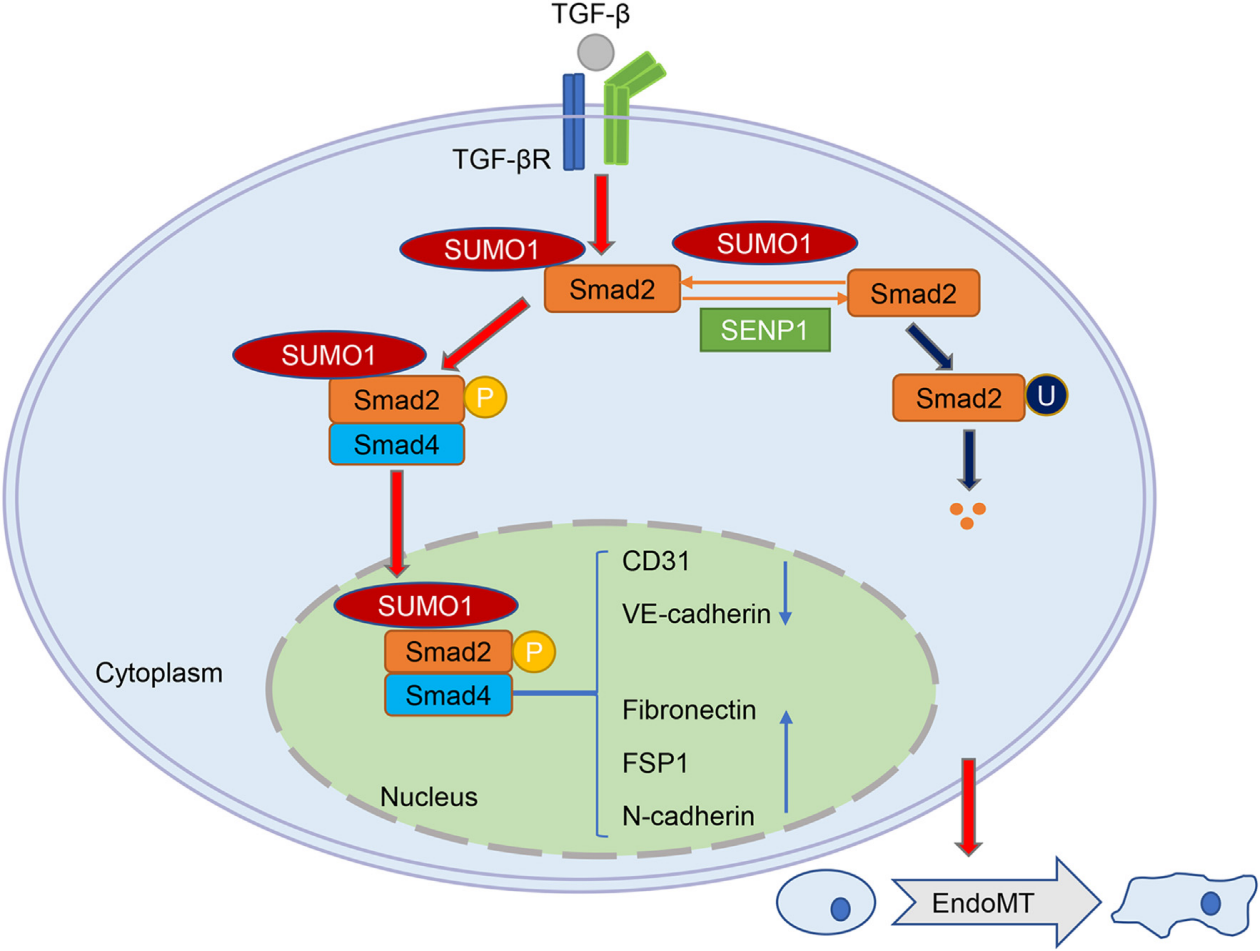
**Schematic diagram of the effect and molecular mechanism of Smad2 SUMOylation in the progression of TGF-β-induced EndoMT.** EndoMT, endothelial–mesenchymal transition; TGF-β, transforming growth factor beta.

Previous studies have shown that TGF-β signaling effectively induces the EndoMT process in ECs. However, whether SUMOylation of Smad2 participates in the EndoMT process and the molecular mechanism remain unclear. Here, we show that Smad2 is predominantly modified by SUMO1 with PIAS2α as the primary E3 ligase, whereas SENP1 mediates the deconjugation of Smad2 SUMOylation. SUMOylation significantly enhances the transcriptional activity of Smad2 and increases the phosphorylation of Smad2 and the formation of the Smad2–Smad4 complex, promoting the nuclear translocation of Smad2. Ultimately, SUMOylation of Smad2 facilitates the EndoMT process in ECs (Fig. 8).

EndoMT is a complex biological process in which ECs are transformed into mesenchymal cells (27). As a critical molecule in the TGF-β signaling pathway, Smad2 is modified by phosphorylation and ubiquitination, and these modifications play an essential role in the TGF-β signaling pathway (28, 29). SUMOylation is an important form of post-translational modification, and to our knowledge, this is the first study to show that Smad2 is SUMOylated and that PIAS2α is the primary E3 ligase responsible for Smad2 SUMOylation (Fig. 1). On the contrary, the SUMOylation of Smad2 is uncoupled by SENP1 (Fig. 3). When the TGF-β signal is activated, the C-terminal SXS motif of Smad2 is phosphorylated, and the activated Smad2 undergoes a conformational change and forms a complex with Smad4, translocates into the nucleus, and regulates gene transcription (30). SUMOylation of Smad2 enhances the protein stability and transcriptional activity of Smad2, thus regulating the transcription levels of downstream genes (Figs. 4 and 5). However, SUMOylation of Smad4 shows no effect on the formation of the Smad2–Smad4 complex, indicating the various effects of SUMOylation on Smad (23). These results indicate the essential roles of Smad2 SUMOylation in TGF-β signaling.

Previous studies have shown that the level of Smad2 phosphorylation increases during EndoMT (31). According to our results, SUMOylation of Smad2 increases its phosphorylation, promotes nuclear translocation, and significantly promotes TGF-β-mediated EndoMT (Figs. 6 and 7). It was demonstrated that SUMOylation of Smad2 is a critical regulator of EndoMT. EndoMT mediated by TGF-β signaling is associated with various diseases, including renal fibrosis and pulmonary hypertension (32). Inhibition of the EndoMT process is a vital way to explore drug targets for atherosclerosis (33) and non–small cell lung cancer (34). Blockade of EndoMT delays the early development of streptozotocin-induced diabetic nephropathy (35). Therefore, targeting Smad2 SUMOylation to regulate TGF-β-mediated EndoMT provides a valid theoretical basis for the clinical treatment of EndoMT-related diseases.

In summary, the present study demonstrates that SUMOylation of Smad2 promotes its phosphorylation and nuclear translocation, increasing its transcriptional activity, and promoting the TGF-β-mediated EndoMT process in ECs. Given the increasing number of pathological diseases associated with EndoMT, the present study lays a foundation for the critical roles of Smad2 SUMOylation in the EndoMT process and provides innovative therapeutic strategies for treating EndoMT-related diseases.

## Experimental procedures

### Cell culture and treatment

HEK293T cells were purchased from the American Type Culture Collection. HUVECs were provided by Dr Yi Zhang (Xi’an Jiaotong University), and HAECs were provided by Dr Baochang Lai (Xi’an Jiaotong University). HEK293T cells were cultured in Dulbecco’s modified Eagle’s medium (DMEM; Gibco), and HUVECs and HAECs were cultured in DMEM/F-12 medium containing 10% fetal bovine serum (Gibco), 100 units/ml penicillin, and 100 μg/ml streptomycin at 37 °C in a 5% CO_2_ atmosphere. The HAEC cells were cultured in DMEM/F-12 medium with additional 10 μg/ml heparin. The siRNA oligonucleotides targeting PIAS2ɑ (sense: AAGAUACUAAGCCCACAUUUGTT, antisense: CAAAUGUGGGCUUAGUAUCUUTT) were purchased from GenePharma. Cells were transiently transfected using Lipofectamine 3000 (Invitrogen), according to the manufacturer’s instructions. For the protein degradation assay, transfected cells were treated with 50 μg/ml CHX at different times, and the whole-cell lysates were analyzed by Western blotting. All experiments in this study were approved by the Committee on the Ethics of Experiments of Shaanxi Normal University.

### Plasmid construction

The eukaryotic expression plasmids HA-SUMO1, HA-SUMO2, HA-PIAS1, HA-PIAS1, HA-PIAS2ɑ, HA-PIAS2β, HA-PIAS3, HA-PIAS4, FLAG-SENP1, catalytic mutant FLAG-SENP1, RGS-SENP2, and RGS-SENP3 have previously been reported (12, 14, 16). The FLAG-Smad2 plasmid was constructed using standard PCR-based strategies with the indicated primers (Table S1), and SUMO site mutants were generated using the Quick-change site-directed mutagenesis kit (TianGen). All plasmids were verified by DNA sequencing.

### Generation of the lentiviral system

The lentiviral expression plasmids pCDH-Smad2 and the SUMO site mutant pCDH-Smad2 were constructed by standard PCR-based strategies with the indicated primers (Table S1). Overexpressed cell lines were generated using a lentiviral system (System Biosciences). The virus was generated in HEK293TN cells by transfecting the packaging (psPAX2) and envelope (pMD2.G) plasmids. Cell culture media were collected 48 h after transfection and immediately transferred to target cells. The transduced cells were selected with puromycin for 48 h. Then 10 ng/ml hTGF-β1 (Cell Signaling) was added to HAECs and HUVECs that stably expressed Smad2w and SUMO mutant Smad2 for 3 days. Real-time PCR or immunoblotting was manipulated to detect the efficacy of overexpression.

### Immunocytochemistry

Cells transfected with the indicated plasmids were grown on coverslips. After 48 h, the cells were washed with PBS, fixed with 4% paraformaldehyde (Sigma), permeabilized with Triton X-100, and then incubated with antibodies. Nonspecific antibody binding was minimized by treatment with 10% goat serum (Yeasen) at room temperature. Primary antibodies were diluted in Triton X-100 and then incubated for 1 h at 37 °C. After that, the cells were washed three times in PBS and then incubated for 1 h at room temperature with secondary Alexa Fluro 488 or 546 fluorophore antibodies (Invitrogen). The cells were then washed three times in PBS, incubated with 4′,6-diamidino-2-phenylindole for 10 min, mounted using antifade mounting solution (Dako), and examined by confocal laser scanning microscopy (Leica).

### Cellular subfractionation

The subcellular fraction protein was isolated by the kit according to the manual (KeyGen). Briefly, FLAG-Smad2 and HA-SUMO1 were transfected into HEK293T cells and cultured for 48 h. All cells were washed and lysed in hypotonic buffer with protease inhibitors (TargetMol), and cell lysates were centrifuged. The supernatants were collected as cytoplasmic extracts, and the pellets were washed three times and lysed with a high salt buffer with protease inhibitors, vortexed, and rotated. Cell lysates were centrifuged, and supernatants were collected as nuclear extracts. Equal amounts of cytoplasmic and nuclear extracts were used for Western blotting.

### RNA isolation and real-time PCR

For quantitative analysis of gene expression, total RNA was extracted using the RNeasy protocol (Qiagen) from cultured cells or transfected cells. RNA was treated with DNase (Promega), and the concentration was determined by measuring the absorbance at 260 nm. Equal amounts of RNA were used to generate complementary DNA using the high capacity complementary DNA reverse transcription protocol (Takara). Quantitative real-time PCR was performed using reaction mixtures of complementary DNA with the indicated primers (Table S2) and SYBR Green reagent (Takara) with the ABI StepOne system (PerkinElmer). PCR was performed in triplicate, and standard deviations representing experimental errors were calculated. All data were analyzed using the ABI PRISM SDS 2.0 software (PerkinElmer). This software allows for the determination of the threshold cycle that represents the number of cycles where the fluorescence intensity is significantly higher than the background fluorescence intensity.

### Western blotting and immunoprecipitation

The transfected cells were extracted and homogenized on ice in lysis buffer with protease inhibitors. Total protein levels were quantified using the BCA assay (Pierce). Equal amounts of protein were separated by electrophoresis and transferred to polyvinylidene difluoride membranes by electroblotting. The membranes were blocked with 5% nonfat dried milk, incubated overnight with primary antibodies (Table S3), washed, and incubated with secondary antibody coupled to peroxidase. Protein levels were detected with a chemiluminescence system (Tanon) after additional washing steps. For immunoprecipitation experiments, cell lysates were incubated overnight with antibodies under denaturing conditions. All incubations were performed at 4 °C with constant agitation. Antibody-bound protein complexes were captured by adding protein A/G agarose and incubating for another 2 h. The protein A/G agarose was pelleted by centrifugation, and the immunoprecipitated protein complex was eluted using SDS-PAGE sample buffer and Western blotting with antibodies.

### Tube formation assay

The prepacked Matrigel (Corning) was melted, added to a precooled 96-well plate, and incubated at 37 °C for 1 h. Then, 20,000 cells were seeded on the plate coated with Matrigel. After incubation at 37 °C, tube formation was observed by an inverted phase-contrast microscope (Leica). The degree of tube formation was quantified by measuring the number of tube-like structures in five randomly chosen fields from each dish.

### Quantification and statistical analysis

All data were presented as the mean ± SEM or SD, and all experiments were performed with at least three repetitions. Differences between groups were evaluated using Student’s *t* test for two-group comparisons and one-way ANOVA followed by Dunnett’s test or Tukey’s test or two-way ANOVA followed by Bonferroni’s test for multiple comparisons among more than two groups. The variance was similar between groups in the statistical comparisons. Statistical significance was defined as *p* < 0.05 (∗*p* < 0.05, ∗∗*p* < 0.01, and ∗∗∗*p* < 0.001).

## Data availability

All relevant data are within the article and its supporting information files.

## Supporting information

This article contains supporting information.

## Conflict of interest

The authors declare that they have no conflicts of interest with the contents of this article.
