# Supplementary material for: SUMOylation of Smad2 mediates TGF-β-regulated endothelial–mesenchymal transition
Source: J Biol Chem. 2023 Sep 9;299(10):105244. doi: 10.1016/j.jbc.2023.105244 (PMC10570702; doi:10.1016/j.jbc.2023.105244)
Supplement: Supporting information [file mmc1.docx]

**Supplemental Tables**

**Table S1. Primers for plasmid construction.**

| Plasmid | Sequence (5’-3’) |
| --- | --- |
| pCDH-Smad2-F | TGCTCTAGAATGTCGTCCATCTTGCCATT |
| pCDH-Smad2-R | ATTTGCGGCCGCTTATGACATGCTTGAGCAAC |
| Smad2-F | CCCAAGCTTATGTCGTCCA TCTTGCCATT |
| Smad2-R | TCCCCCGGGTTATGACATGCTTGAGCAAC |
| Smad2 K156R-F | TCTTAGGAAGGATGAAGTATGTGTAAACCCTTACCAC |
| Smad2 K156R-R | CTTCATCCTTCCTAAGATTAAAAGCATATTCGCAGTTTTCAATTGC |
| Smad2 K383R-F | TGTAATCTGAGGATCTTCAACAACCAGGAATTTGCTGCTCTT |
| Smad2 K383R-R | GTTGAAGATCCTCAGATTACAGCCTGGTGGAATTTTACACA |

**Table S2. Primers for quantitative real-time PCR.**

| Primer | Sequence (5’-3’) |
| --- | --- |
| 18s-F | CACGGACAGGATTGACGAAT |
| 18s-R | CGAATGGGGTTCAACGGGTT |
| E-cadherin-F | CAGCACGTACACAGCCCTAA |
| E-cadherin-R | GCTGGCTCAAGTCAAAGTCC |
| p15-F | CCAGATGAGGACAATGAG |
| p15-R | AGCAAGACAACCATAATCA |
| Snail-F | GGGCAGGTATGGAGAGGAAGA |
| Snail-R | TTCTTCTGCGCTACTGCTGCG |
| ZEB1-F | AGGCATATGGTGACGCACAA- |
| ZEB1-R | CTTGAACTTGCGGTTACCTGC |

**Table S3. Antibodies for Western blotting and co-IP assay.**

| Antibody | Identifier | Source |
| --- | --- | --- |
| α-SMA | 14385-1-AP | Proteintech |
| CD31 | 11265-1-AP | Proteintech |
| Fibronectin | 15613-1-AP | Proteintech |
| Flag | [F1804](https://www.sigmaaldrich.cn/CN/zh/product/sigma/f9291?context=product) | Sigma |
| FSP1 | 16105-1-AP | Proteintech |
| HA | 3724 | Cell Signaling Technology |
| N-cadherin | 22018-1-AP | Proteintech |
| p-Smad2 | 3108 | Cell Signaling Technology |
| SENP1 | 11929 | Cell Signaling Technology |
| SENP2 | ab58418 | Abcam |
| SENP3 | 5591 | Cell Signaling Technology |
| Smad2 | 12570-1-AP | Proteintech |
| Smad3 | 66516-1-lg | Proteintech |
| Smad4 | 46535 | Cell Signaling Technology |
| SUMO1 | 4930 | Cell Signaling Technology |
| SUMO2/3 | 4971 | Cell Signaling Technology |
| VE-cadherin | 2500 | Cell Signaling Technology |
